# Diagnostic value of whole-body MRI in Opsoclonus-myoclonus syndrome: a clinical case series (3 case reports)

**DOI:** 10.1186/s12880-019-0372-y

**Published:** 2019-08-20

**Authors:** Corinna Storz, Roland Bares, Martin Ebinger, Rupert Handgretinger, Ilias Tsiflikas, Jürgen F. Schäfer

**Affiliations:** 1 Division of Pediatric Radiology, Department of Diagnostic and Interventional Radiology, University Hospital Tuebingen, Hoppe-Seyler Str. 3, 72076 Tuebingen, Germany; 2 Department of Nuclear Medicine and Clinical Molecular Imaging, University Hospital Tuebingen, Tuebingen, Germany; 3 Department of Pediatric Surgery, Children’s University Hospital Tuebingen, Tuebingen, Germany; 4 Department of Hematology/Oncology, Children’s University Hospital Tuebingen, Tuebingen, Germany

**Keywords:** Whole-body MRI, Scintigraphy, Neuroblastoma, Opsoclonus-myoclonus syndrome

## Abstract

**Background:**

Opsoclonus-myoclonus syndrome (OMS) is a rare clinical disorder and typically occurs in association with occult neuroblastic tumor in pediatric patients. I-123 metaiodobenzylguanidine (mIBG) scintigraphy is widely adopted as screening procedure in patients with suspected neuroblastic tumor. Also, contrast-enhanced magnetic resonance imaging (MRI) or computed tomography (CT) are involved in the imaging workup, primarily for the assessment of the primary tumor region. However, the diagnostic value of whole-body MRI (WB-MRI) for the detection of occult neuroblastic tumor in pediatric patients presenting with OMS remains unknown.

**Case presentation:**

We present three cases of patients with OMS, in whom WB-MRI revealed occult neuroblastic tumor masses, whereas scintigraphy was inconclusive:

In a 17 months old girl with OMS, WB-MRI revealed a paravertebral mass. After thoracoscopic resection, histopathology revealed a ganglioneuroblastoma.

A 13 months old boy presenting with OMS WB-MRI detected a tumor of the left adrenal gland; histopathology demonstrated a ganglioneuroblastoma after adrenalectomy.

In a 2 year old boy with OMS, immunoscintigraphy at the time of diagnosis was inconclusive. At the age of 13 years, a WB-MRI was performed due to persistent neurological symptoms, revealing a paravertebral retroperitoneal mass, which was classified as ganglioneuroblastoma.

**Conclusion:**

In OMS, particularly in the setting of inconclusive scintigraphy, WB-MRI may be considered as a valuable alternative in the early phase of diagnostic work-up.

## Background

Opsoclonus-myoclonus syndrome (OMS), also known as dancing eye syndrome or opsoclonus-myoclonus-ataxia syndrome, is an extremely rare autoimmune neurological disorder, with an incidence of approximately 0.18 cases per 1.000.000 in the UK population annually [1]. OMS affects mainly young children with a typical onset at 18 months of age and typically presents subacutely with jerky unsteadiness and ataxia as well as intermittent ocular flutter or opsoclonus with rapid, multidirectional eye movements [1, 2]. As almost half of the pediatric patients with OMS are associated with an underlying neuroblastic tumor (e.g. neuroblastoma, ganglioneuroblastoma, ganglioneuroma), and, conversely, approximately 2–3% of the pediatric patients with neuroblastoma present with OMS, it is defined as a paraneoplastic neurological disorder [1]. Previous studies presumed, that there might be an immune-mediated encephalopathy caused by a cross-reactive autoimmune reaction between neuroblastoma cells and the central nervous system and a variety of antibodies have been described, for example IgM and IgG antibodies to neural tissues and antigens such as components of Purkinje cells, however, the detailed pathogenesis and epidemiology remains unclear [3, 4].

Interestingly, patients with coincident OMS and neuroblastoma have a favorable survival and non-metastatic disease [2, 5, 6]. However, there is also research indicating that a delayed diagnosis of OMS may result in late neurological and neuropsychological sequelae. Especially children with young age at disease onset and children with severe initial symptoms are postulated to be at significant risk of developing long-term neurological sequelae such as cognitive deficits [7, 8]. Furthermore, a delayed diagnosis of OMS was found to be associated with neurological and neuropsychological sequelae, thus, the role of early diagnosis of OMS and possibly underlying neuroblastic tumors is of critical importance [7].

Beside clinical examination and laboratory tests such as the urinary catecholamine excretion, I-131 and I-123 metaiodobenzylguanidine (mIBG) scintigraphy provides high sensitivity and specificity for the detection of neuroblastoma and its metastases and has been adopted widely as a screening procedure and for disease monitoring in pediatric patients with neuroblastic tumor [9–13]. However, there is also very early evidence that computed tomography (CT) and magnetic resonance imaging (MRI) are valuable tools for the detection of primary tumor in patients with OMS [14, 15].

In this case series we report on three pediatric patients presenting with OMS and a negative I-123-mIBG scintigraphy, in whom whole-body MRI (WB-MRI) correctly identified neuroblastic tumor sites.

## Case presentations

### Case 1

**Fig. 1:**
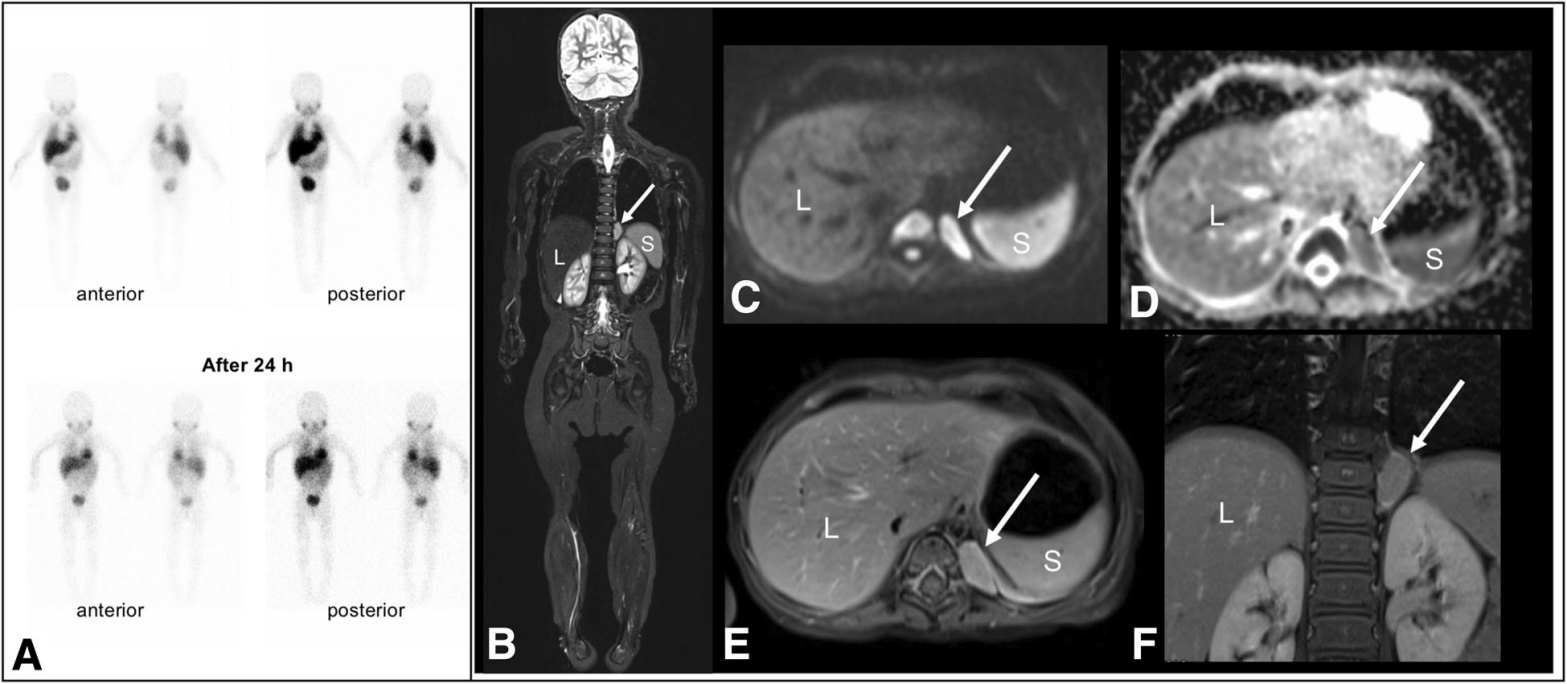
Case 1: 17 months old girl presenting with opsoclonus-myoclonus syndrome. Whole-body I-123-mIBG scintigraphy with 60 mBq I-123-mIBG did not reveal any pathological tracer uptake (**a**). WB-MRI revealed a solid left-sided paravertebral mass extending to the correlated neuroforamina (*white arrow*) at the level of thoracic vertebrae T 9/10 with signal hyperintensity in T2 weighted Turbo-Inversion Recovery-Magnitude (TIRM) sequences (**b**). In diffusion weighted imaging, correlated restricted diffusion could be detected with low ADC (**c** and **d**) and strong enhancement with hyperintensity could be detected in the T1 weighted sequence after the administration of 2 ml gadolinium compound (**e** and **f**). *L = liver, S = spleen*

A 17 months old girl presented with trembling voice, opsoclonus, head tremor, unsteadiness and ataxia for one month (Table 1). Routine laboratory results demonstrated a leukocytosis (22,850 cells per μl) while all other parameters were within normal limits. Abdominal ultrasound was unremarkable. Based on clinical presentation the patient was suspicious for a neuroblastic tumor and a WB-MRI was performed one day after the presentation (Fig. 1). WB-MRI revealed a left-sided paravertebral mass at the level of thoracic vertebrae T 9/10 (2.6 × 1.1 × 2.2 cm), hyperintense in T1 and T2 weighted sequences with low ADC in in diffusion imaging, and strong diffuse enhancement after the administration of gadolinium compound. I-123-mIBG scintigraphy did not reveal any non-physiological tracer uptake. Following thoracoscopic resection of the paravertebral mass, histopathology showed matured neuroblastoma tumor cells with schwannian stroma, consistent with a thoracic ganglioneuroblastoma (Hughes classification: grade 1a). Consequently, treatment with immunoglobulins, steroids, cyclophosphamide and rituximab was initiated. Her regular follow-up examinations were unremarkable without any neurological sequelae.

**Table 1:** Demographical, clinical and neurological findings as well as imaging findings and histopathological results in the patients presenting with opsoclonus-myoclonus syndrome

	Age at presentation	Gender	Chief complaint	Abdominal ultrasound	Whole-body MRI	Whole-body Scintigraphy	Tumor localization	Pathology/Diagnosis
Case 1	17 months	female	opsoclonus, head tremor, unsteadiness, ataxia	negative	positive	negative	left-sided paravertebral T9/10	thoracal ganglioneuroblastoma
Case 2	13 months	male	opsoclonus, ataxia, unsteadiness	negative	positive	negative	left adrenal gland	adrenal ganglioneuroblastoma
Case 3	13 years 11 months	male	opsoclonus, persistent progressive resting and postural tremor, ataxia	not performed	positive	negative	right-sided paravertebral T11-L1	retroperitoneal ganglioneuroma

### Case 2

**Fig. 2:**
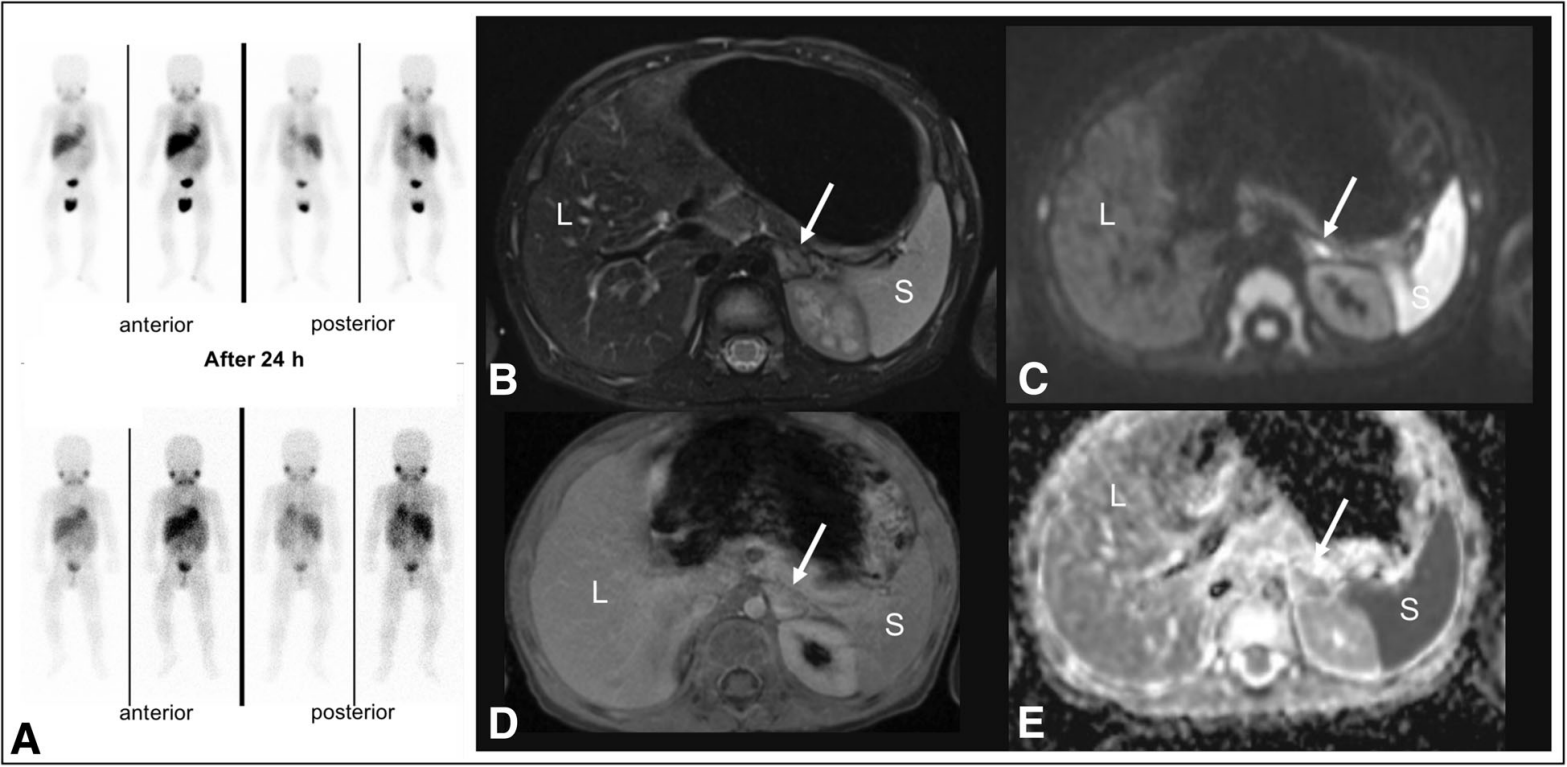
Case 2: 13 months old boy presenting with opsoclonus-myoclonus syndrome. No pathological tracer uptake could be detected in the I-123-mIBG scintigraphy with 40 mBq I-123-mIBG (**a**). WB-MRI revealed a T2 hyperintense mass (*white arrow*) of the left adrenal gland (**b**) with diffuse enhancement in the post-contrast T1 weighted sequences after the administration of 2 ml gadolinium compound (**d**). Restricted diffusion in diffusion imaging (**c**) with partial low ADC (**e**) could be detected. *L = liver, S = spleen*

A 13 months old boy was referred to the department with neurological conspicuities presenting multiple relapses, failure to walk and speak as well as intermittent opsoclonus for a few months (Table 1). Abdominal ultrasound examination revealed no abnormal finding. Clinical examination confirmed OMS, laboratory testing showed a slightly increase of lymphocytes (48,000 cells per μl). Urinary catecholamine excretion revealed no pathological increase. In order to rule out neuroblastic neoplasia, an I-123 MIBG scintigraphy was performed, which was negative. Given the persistent clinical suspicion, a WB-MRI was performed (Fig. 2), showing a triangular T2-hyperintense mass (1.0 × 1.1 cm) adjacent to the upper pole of the left kidney with mild enhancement in the post-contrast sequences and partially restricted diffusion in the diffusion weighted MRI consistent with a neuroblastic tumor to the left adrenal gland. Following an adrenalectomy, histopathology revealed fibro-muscular tissue infiltrated by neuroblastic tumor nests with focal ganglionic differentiation and adjoining differentiated ganglioneuroma-like schwannian stroma, consistent with an intermixed ganglioneuroblastoma of the left adrenal gland (Shimada system: schwannian stroma-rich ganglioneuroblastoma, intermixed type). Steroid therapy, immunoglobulins, corticotropin and rituximab were initiated for approximately 2 years. In regular follow-up examinations including whole body MRI, no recurrence was detected and urinary tests were negative. Furthermore, neurological anomalies and OMS completely regressed.

### Case 3

**Fig. 3:**
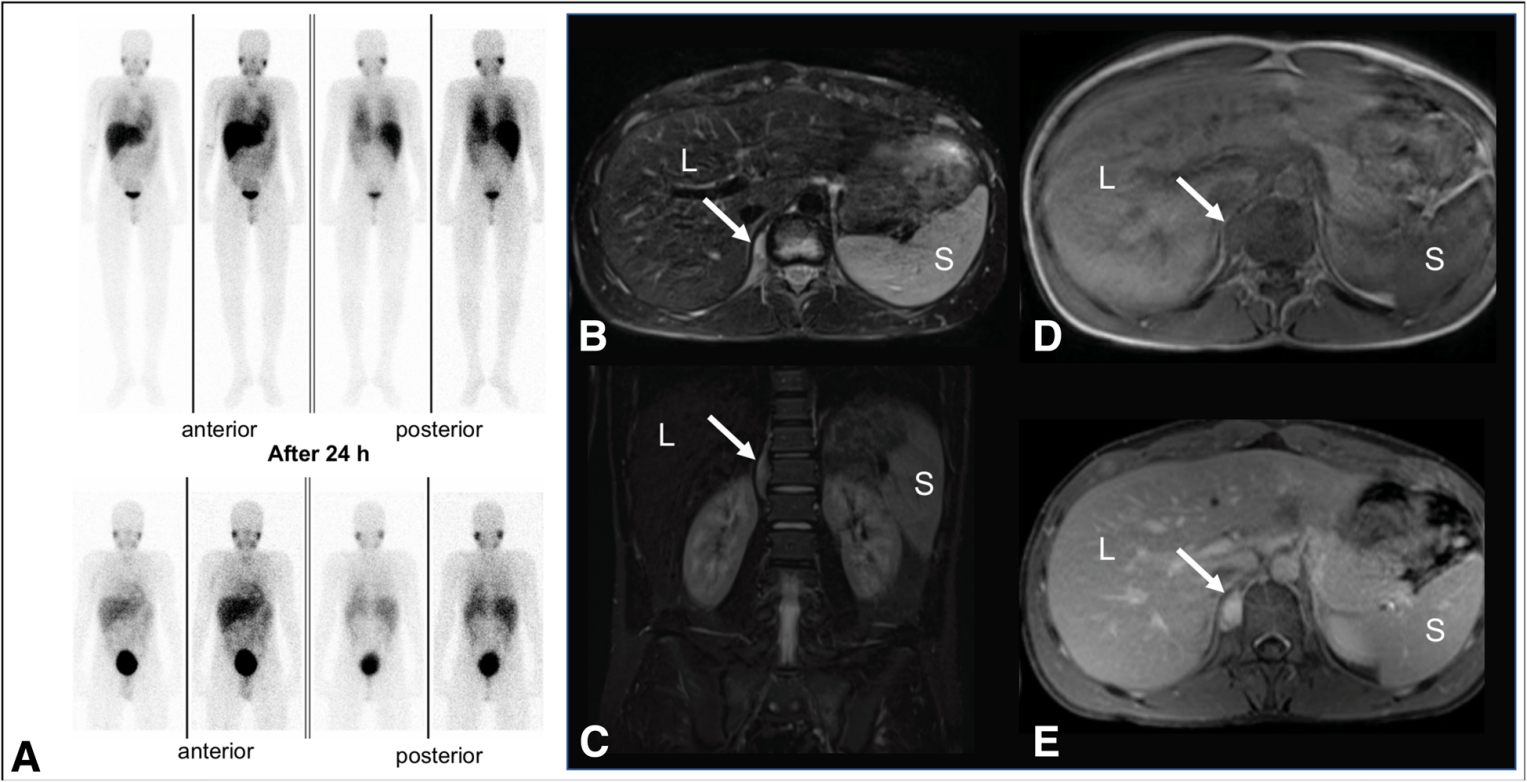
Case 3: 13 year old boy presenting with steroidresponsible opsoclonus-myoclonus syndrome. No I-123 MIBG tracer uptake could be detected in scintigraphy after the administration of 108 mBq I-123-mIBG (**a**). WB-MRI revealed a paravertebral right-sided retroperitoneal mass (*white arrow*) at the level of T11 to L1 with hyperintensity in T2 weighted sequences (**b**, axial T2w and **c**, coronal) and contrast enhancement in the post-contrast T1 weighted sequence after intravenous injection of 7 ml gadolinium compound (**d**, native T1 weighted sequence and E, post-contrast T1 weighted sequence). *L = liver, S = spleen*

A 2 year old boy presented with steroidresponsible OMS and progressive resting and postural tremor and ataxia (Table 1). Laboratory testing was within normal range. A 99 m-Tc-Anti-GD2 immunoscintigraphy, performed at the time of diagnosis, was negative. Annual follow-up examinations including recurring clinical examinations, laboratory tests, abdominal ultrasound and urinary catecholamine excretion tests were negative. Due to the persistent neurological symptoms, a WB-MRI was performed at the age of 13 years to preclude a paraneoplastic genesis associated with OMS (Fig. 3). The WB-MRI demonstrated a paravertebral right-sided retroperitoneal mass at the level of T11 to L1 (2.9 × 1.5 × 5.4 cm), remarkable hyperintensity in T2 and vigorous contrast enhancement. Complementing I-123-mIBG scintigraphy revealed no abnormality. Following laparoscopic resection, the histopathology demonstrated mature ganglion cells and fibrillary stroma with spindle-shaped cells, consistent with benign ganglioneuroma (Shimada system: schwannian stroma-dominant ganglioneuroma, mature subtype). Intermittent steroid therapy was continued for treatment of residual OMS. In regular follow-up examinations, the patient still presented neurological features and residual OMS, which responded well to steroid therapy.

## Discussion and conclusions

OMS is a rare pediatric disease, which poses significant challenge to the caregiving physicians. We report on three patients who presented with OMS due to an underlying neuroblastic tumor. In all cases, abdominal ultrasound and I-123-mIBG scintigraphy were unremarkable while WB-MRI identified small, but characteristic lesions consistent with neuroblastic masses. While routine diagnostic work-up does not comprise additional imaging procedures, conclusive WB-MRI was performed due to persistent clinical suspicion, which highlights the valuable role of WB-MRI in the detection of occult neuroblastic tumors in patients with OMS.

Due to its very high sensitivity and specificity derived from previous research, mIBG scintigraphy is a robust and well-established screening modality for the detection of primary tumor site as well as lymph node involvement and metastases in pediatric patients with suspected neuroblastic tumor [9–13]. Specifically, in a large Cochrane Database review including 11 studies, Bleeker et al. reported a sensitivity of I-123-mIBG scintigraphy ranging from 68 to 100% in patients with histologically proven neuroblastoma [9]. However, there is also evidence of false-positive results and 10% of neuroblastomas are associated with a negative mIBG uptake [9, 11, 16]. The false-negative results of I-123-mIBG scintigraphy in our cases cannot be fully explained. The relative small tumor size as well as missing receptor-uptake of the lesions, for example in non-secreting low grade neuroblastoma may be potential causes [9, 11, 16, 17]. In addition, due to the physiological mIBG uptake of the adrenal glands, the detection of tumor lesions located close to the kidney and adrenal glands might be significantly limited. Reuland et al. found that 99 m-Tc-Anti-GD2 immunoscintigraphy might be a useful addition for diagnosis of new tumor lesions superior to I-123-mIBG scintigraphy in high grade neuroblastoma due to an earlier uptake of neuroblastoma lesions compared to I-123-mIBG scintigraphy [18]. However, negative 99 m-Tc-Anti-GD2 immunoscintigraphy in our case #3 may be explained by present low-grade neuroblastic tumor genesis, resulting in a delayed detection of neuroblastic tumor.

In contrast, regarding the manifestation of OMS as a paraneoplastic symptom of possibly underlying neuroblastic tumor, recent studies found cross sectional imaging by CT or MRI to be promising and sensitive tools in the setting of detecting the primary tumor site [14, 15, 19]. As such, MRI has been classified as equal to I-123-mIBG scintigraphy, which remains the mandatory imaging modality in the primary tumor imaging workup in neuroblastoma [20]. However, the major drawbacks of I-123-mIBG scintigraphy are the extensive procedure, limited spatial resolution as well as the radiation exposure accompanied by the intravenous injection of radioactive I-123, which is additionally associated with size and administered activity of the lesion [21].

CT, as a widely available modality, allows for fast acquisition and reduces sedation time. Razek et al. showed, that whole-body CT provides a high detection rate for cortical and medullary bone lesions, spinal fracture and extraosseous spinal lesions [22]. However, whole-body CT is associated with significant radiation exposure, which is of particular concern in the pediatric patient population.

WB-MRI is diffusing increasingly clinical routine in pediatric radiology, especially due to technical developments and accelerated acquisition time as well as the high anatomic resolution. Apart from several imaging protocol recommendations, WB-MRI has shown to be a precise and comprehensive image modality for children suffering from solid tumors. Furthermore, diffusion-weighted MRI provides further information about tumor characterization at a cellular level and was found to be a beneficial tool in the differentiation of paraspinal benign versus malignant neurogenic tumors [23]. Moreover, due to the high cellular density, neuroblastoma frequently demonstrates diffusion restriction, hence, diffusion-weighted imaging is a critical part of the MRI protocol in the imaging work-up of patients with suspected neuroblastoma [24].

Previous studies reported promising initial experiences with 68Ga-DOTA-Octreotate positron emission tomography (PET) and simultaneous CT in patients with OMS [25]. This is in line with the observation that hybrid imaging modalities such as PET/CT or non-ionizing PET/MRI are emerging as key diagnostic modalities in the work-up of oncologic pediatric patients [26]. This is further accentuated as novel tracers, for instance 18 F-Meta fluorobenzyl guanidine as a PET analog of mIBG, seem to be promising markers in the diagnostic setting of neuroendocrine tumors with the advantage of higher tracer uptake, higher spatial resolution, and reduced radiation exposure in comparison to mIBG [27].

Our case series and review of the literature emphasize the value of WB-MRI in occult neuroblastic tumors, particularly in the setting of a negative I-123-mIBG scintigraphy that was present in all cases. In these patients, MRI detected the primary tumor site and arrived at the diagnosis without ionizing radiation. Our case series indicates, that WB-MRI enables a rapid diagnosis of neuroblastic tumor sites and may thus improve current diagnostic pathways and impede delay in treatment. Thus, further prospective, well-designed studies comparing WB-MRI and scintigraphy in the assessment of occult tumor manifestations in pediatric population is strongly warranted and justified. Given its non-ionizing nature, it may thus represent a favorable alternative for early diagnosis of possibly underlying neuroblastic tumors in the work-up of children with occult neuroblastic tumors, especially in cases with negative mIBG scintigraphy. However, more scientific evidence is needed, particularly in comparison with I-123-mIBG scintigraphy.

## Data Availability

The datasets used and/or analyzed during the current study are available from the corresponding author on reasonable request.

## Abbreviations

CT: Computed tomography
mIBG: Metaiodobenzylguanidine
MRI: Magnetic resonance imaging
OMS: Opsoclonus-myoclonus syndrome
